# Increased Mortality Risk for Cancers of the Kidney  and Other Urinary Organs among Chinese Herbalists

**DOI:** 10.2188/jea.JE20080035

**Published:** 2009-01-30

**Authors:** Hsiao-Yu Yang, Jung-Der Wang, Tsai-Chang Lo, Pau-Chung Chen

**Affiliations:** 1 Institute of Occupational Medicine and Industrial Hygiene, National Taiwan University College of Public Health, Taipei, Taiwan; 2 Department of Occupational Medicine, Buddhist Tzu Chi General Hospital, Hualien, Taiwan; 3 Department of Internal Medicine, and Department of Environmental and Occupational Medicine, National Taiwan University Hospital, Taipei, Taiwan; 4 Miaoli County Public Health Bureau, Miaoli, Taiwan

**Keywords:** Chinese herbal drugs, Chinese herbalist, aristolochic acid, chronic kidney disease, urological cancer

## Abstract

**Background:**

A national survey in Taiwan has shown that Chinese herbal therapy increases the risk of chronic kidney disease. However, it is unknown whether herbal therapy will increase the risk of urological cancers. The purpose of this study was to determine whether Chinese herbalists are at higher risk for urological cancers.

**Methods:**

We studied all Chinese herbalists in Taiwan that were registered in the Chinese Herbalist Labor Union between 1985 and 2000. We retrospectively followed their survival status and causes of death using the National Mortality Registry Database from 1985 to 2004. Standardized mortality ratios (SMRs) for urological cancers in herbalists were calculated and compared with those of the general population of Taiwan.

**Results:**

A total of 6548 Chinese herbalists were enrolled and 88,289 person-years were accrued during the observation period. After adjustment for age and sex, the SMR for urological cancers was significantly higher for Chinese herbalists than for the general population (SMR = 3.10; 95% CI: 1.41–5.87). When further stratified by location, the SMR for kidney cancer and other urinary organ cancers (SMR = 3.81; 95% CI: 1.39–8.28) except bladder cancer (SMR = 2.26; 95% CI: 0.47–6.59) were significantly higher for the Chinese herbalists. The SMR for chronic and unspecified nephritis, renal failure, and renal sclerosis were also significantly higher for herbalists (SMR = 2.40; 95% CI: 1.40–3.84).

**Conclusions:**

Chinese herbalists have a significantly higher risk for urological cancers. This increased risk among herbalists highlights the urgent need for safety assessments of Chinese herbs.

## INTRODUCTION

Chinese herbal drugs are widely used all over the word, especially in East Asia and Europe.^1^^–^^3^ An important reason for their extensive use is that people believe herbal drugs to be mild and harmless.^4^ In Taiwan, traditional Chinese medicine is considered to be an integral part of the health care system.^5^^,^^6^ Recently however, many cases of nephropathy and renal failure related to the use of Chinese herbs have been reported in Taiwan.^7^^–^^9^ There is also increasing evidence that the use of Chinese herbal drugs may carry some health risks, especially to the kidney.^7^^,^^10^^–^^12^

Taiwan has the highest incidence of end-stage renal disease (ESRD) in the world, even though the leading causes of ESRD—diabetes and hypertension—are not as prevalent as elsewhere.^13^ The high prevalence of chronic kidney disease (CKD) in Taiwan may substantially contribute to the high incidence and prevalence of ESRD.^14^ Although many factors may contribute to the development of CKD in Taiwan, clinical observations point to the widespread use of Chinese herbal drugs as one of the main causes.^7^ A national survey in Taiwan conducted from 1993 to 1996 found that use of herbal therapy was an independent risk factor for CKD.^15^ However, it is unknown whether the use of herbal therapy increases the risk for urological cancers.

Chinese herbalists constitute a special occupational cohort in Taiwan. They work in traditional Chinese herbal stores, where they acquire, process, and sell herbs. They do not attend medical school, but instead inherit their knowledge of Chinese medicine by means of a “master and apprentice” system that has been passed down from generation to generation. Because of their deeply rooted belief in Chinese medicine, Chinese herbalists seldom treat patients with Western medicines. They generally use Chinese herbal drugs to treat all illnesses because they think herbal drugs are mild and harmless. Based on the theory of Yin–Yang balance in Chinese medicine,^16^^–^^18^ herbalists also take Chinese herbal drugs as tonics to improve their well-being. Chinese herbalists in Taiwan are more likely to ingest Chinese herbal drugs than the general population.^6^^,^^19^ The purpose of this study was to determine whether Chinese herbalists are at higher risk for urological cancers.

## METHODS

We compared the mortality of Chinese herbalists and the general population in Taiwan. Date of birth, sex, and employment history were obtained from the database of the Bureau of Labor Insurance. In Taiwan, the Labor Insurance Program came into effect in 1960. It is a compulsory social insurance program that all workers aged 15–60 are required to join. The Chinese Herbalist Labor Union was established in 1985, and all herbalists who work in traditional Chinese herbal stores are required to join. In this study, we enrolled all Chinese herbalists who had joined the Chinese Herbalist Labor Union and were registered with the Bureau of Labor Insurance between 1985 and 2000. Any case of coding error in the database, eg, the date of death was earlier than the date of last employment, was excluded from further analysis.

Because Chinese herbalists seldom employ Western medicine, and have greater access to herbal drugs, we assumed that herbalists employed in herbal stores were more likely than the general population to ingest Chinese herbal drugs. The duration of exposure to Chinese herbal drugs was defined as the date of registration with the Chinese Herbalist Labor Union to either the date of departure from the union for any reason or the end of the observation period. Survival status, date of death, and cause of death were obtained by data linkage with the Taiwan National Mortality Registry Database, which has been a comprehensive household registration system since 1952. The cause of death on death certificates, however, has been completely computerized and coded in ICD-9 only since 1985.^20^

For the purpose of international comparison, we converted ICD-9 to ICD-10.^21^ The observation period was from 1985 to the end of 2004. The study was approved by the Ethics Committee of the National Taiwan University College of Public Health.

We used the PC Life Table Analysis System (LTAS)^21^ Version 1.0d developed by the National Institute for Occupational Safety and Health (NIOSH) to calculate the standardized mortality ratio (SMR) for each cause of death among Chinese herbalists in comparison with the general population of Taiwan. The observed rates for the herbalists were compared with the rates from a referent population via indirect standardization. The indirect standardization was performed by comparing observed deaths within every 5-year stratum with expected deaths, where expected deaths were computed by multiplying the referent sex-, age-, and calendar-time-specific mortality rates of the general population in Taiwan by the observed person-years at risk in each stratum. The observed and expected deaths were then summed across all strata. This was done for each of the cause of death categories, and the SMRs were then calculated by dividing total observed deaths (numerator) by total expected deaths (denominator). The 95% confidence interval and two-sided *P*-values were calculated under the assumption that the observed deaths followed a Poisson distribution. Because exposure requires a minimum induction period to cause an effect, it is usually necessary to assign a lag period in calculating SMR, and the person-years and deaths accrued within the lag period will be placed into the unexposed group.^21^ In this study, sensitivity analysis with 0-, 5-, and 10-year lag periods was performed.

## RESULTS

The cohort consisted of 6555 Chinese herbalists. After excluding 7 cases with coding errors, this study finally enrolled 6548 herbalists for analysis, including 3088 male and 3460 female. A total of 41,441 male person-years and 46,848 female person-years were accrued during the observation period. Table 1 shows the general mortality risks among herbalists. Although the SMRs for death from all causes and death from all cancers were not higher among herbalists, the SMR for urological cancers was significantly higher (SMR = 3.10; 95% CI: 1.41–5.87). When we further stratified urological cancers by location, a significantly higher SMR among herbalists was noted for kidney and other urinary organ cancers (SMR = 3.81; 95% CI: 1.39–8.28) except bladder cancer, for which the difference in SMR was not significant (SMR = 2.26; 95% CI: 0.47–6.59).

**Table 1.** Standardized mortality ratios (SMR) by cause of death among Chinese herbalists

		**Male**			**Female**		
			
Cause of death	ICD-10	O/E	SMR	95% CI	O/E	SMR	95% CI
MN of lip, buccal cavity, &	C00-14,C46.2,	13/10.75	1.21	0.64-2.07	1/1.85	0.54	0.01-3.00
pharynx							
MN of digestive organs &	C15-26,C48	40/40.98	0.98	0.70-1.33	15/15.62	0.96	0.54-1.58
peritoneum							
MN of respiratory system	C30-34,C37-38,	6/14.5	0.41*	0.15-0.90	11/6.67	1.65	0.82-2.95
MN of breast	C50	-	-	-	8/7.16	1.12	0.48-2.20
MN of male genital organs	C61-62	2/0.82	2.44	0.30-8.82	-	-	-
MN of female genital organs	C51-58	-	-	-	5/6.87	0.73	0.24-1.70
MN of urinary organs	C64-68	4/1.90	2.10	0.57-5.37	5/1.00	4.99**	1.62-11.66
Neoplasms of lymphatic &	C46.3,C81-85,C88,C90-96	6/3.79	1.58	0.58-3.45	3/2.58	1.16	0.24-3.40
hematopoietic tissue							
Diabetes mellitus	E10-14	15/12.81	1.17	0.66-1.93	7/10.48	0.67	0.27-1.38
Diseases of the blood &	D46.0-46.4,D46.7-46.9,	1/0.45	2.22	0.06-12.32	0/0.39	0	0.00-9.39
blood-forming organs	D50-75,D77,D89,I88,R72						
Diseases of the nervous system &	E75,R44,G08,G10-31,	1/2.65	0.38	0.01-2.09	1/1.51	0.66	0.02-3.67
sense organs	G35-43,G45,G47,						
	G50-93,G95-H02,						
	H04-93,H95						
Diseases of the heart	I00-09,I11,I13,I20-22,	26/21.66	1.20	0.78-1.75	4/8.71	0.46	0.13-1.17
	I24-25,I51,I30-38,I40,						
	I42,I44-52,I97,R00.1,						
	R00.8						
Diseases of the respiratory system	J00-06,J10-18,J20-22,	12/11.87	1.01	0.52-1.77	3/4.47	0.67	0.14-1.96
	J30-47,J60-64,J66-95,						
	J98-99,R09.1,A48.1						
Diseases of the digestive system	K00-22,K25-31,K35-38,	28/30.01	0.93	0.62-1.34	8/8.18	0.98	0.42-1.93
	K40-46,K50-66,K70-76,						
	K78-92,R68.2						
Diseases of the genitourinary	N00-07,N10-21,N25-28,	9/5.51	1.63	0.75-3.10	9/4.48	2.01	0.92-3.81
system	N30-32,N34-36,N39-50,						
	N60-64,N70-99,R31						
All cancers		73/76.31	0.96	0.75-1.20	49/44.47	1.10	0.82-1.46
All deaths		254/260.86	0.97	0.86-1.10	125/124.70	1.00	0.83-1.19

ICD-10: International Classification of Diseases, 10th Revision.

MN: malignancy.

O: Observed number of deaths.

E: Expected number of deaths.

CI: confidence interval.

*: two-tailed *P* < 0.05, **: two-tailed *P* < 0.01.

The risk for urological cancers appeared to differ by sex. As compared to the general population, the SMR for urological cancers was significantly higher in female herbalists (SMR = 4.99; 95% CI 1.62–11.66), but not in male herbalists (SMR = 2.10; 95% CI: 0.57–5.37). When we further stratified urological cancers by location, the SMR for kidney and other urinary organ cancers was significantly higher in female herbalists (SMR = 6.48; 95% CI: 1.77–16.58), but not in male herbalists (SMR = 2.09; 95% CI: 0.25–7.53). These findings are summarized in Table 2. Sensitivity analysis showed that the mortality risks for urological cancers were all significantly increased with 0-, 5-, and 10-year lag periods, as summarized in Table 3.

**Table 2.** Standardized mortality ratios (SMR) by type of malignant neoplasm of the urinary organs or kidney disease, stratified by sex

		Observed	Expected		
Cause of death	ICD-10	deaths	deaths	SMR	95% CI
**Male**					
Malignant neoplasm of urinary organ	C67-C68	4	1.90	2.10	0.57-5.37
Malignant neoplasm of kidney & other urinary organs	C64-66,C68	2	0.96	2.09	0.25-7.53
Malignant neoplasm of bladder	C67	2	0.95	2.12	0.26-7.64
Acute glomerulonephritis, nephritic syndrome, renal failure	N00,N01.0-01.8,	0	0.89	0.00	0.00-4.15
	N04,N14.4,N17				
Chronic and unspecified nephritis, renal failure, renal sclerosis	N01.9,N03,N05,	9	3.82	2.36*	1.08-4.48
	N07,N14.0-14.3,				
	N15-16,N18-19,N26				
**Female**					
Malignant neoplasm of urinary organ	C67-C68	5	1.00	4.99**	1.62-11.66
Malignant neoplasm of kidney and other urinary organs	C64-66,C68	4	0.62	6.48**	1.77-16.58
Malignant neoplasm of bladder	C67	1	0.38	2.60	0.07-14.44
Acute glomerulonephritis, nephritic syndrome, renal failure	N00,N01.0-01.8,	1	0.44	2.26	0.06-12.57
	N04,N14.4,N17				
Chronic and unspecified nephritis, renal failure, renal sclerosis	N01.9,N03,N05,	8	3.27	2.45*	1.05-4.82
	N07,N14.0-14.3,				
	N15-16,N18-19,N26				

ICD-10: International Classification of Diseases, 10th Revision.

CI: confidence interval.

*: two-tailed *P* < 0.05, **: two-tailed *P* < 0.01.

**Table 3.** Standardized mortality ratios (SMR) and 95% confidence intervals by malignant neoplasm of urinary organs or kidney disease among Chinese herbalists, stratified by sex and lag period

		0-year lag		5-year lag		10-year lag	
				
Cause of death	ICD-10	deaths	SMR (95% CI)	deaths	SMR (95% CI)	deaths	SMR (95% CI)
**Male and female**							
MN of urinary organ	C67-C68	9	3.10**(1.41-5.88)	8	3.32**(1.43-6.54)	7	4.47**(1.79-9.20)
MN of kidney & other	C64-66,C68	6	3.81**(1.39-8.29)	5	3.82*(1.24-8.93)	4	4.71*(1.28-12.04)
urinary organs							
MN of bladder	C67	3	2.26(0.47-6.59)	3	2.72(0.56-7.95)	3	4.18(0.86-12.23)
Acute glomerulonephritis,	N00,N01.0-01.8,N04,	1	0.75(0.02-4.18)	1	0.94(0.02-5.20)	0	-
nephritic syndrome, renal	N14.4,N17						
failure							
Chronic and unspecified	N01.9,N03,N05,N07,	17	2.40**(1.40-3.84)	11	1.82(0.91-3.26)	7	1.76(0.70-3.62)
nephritis, renal failure, renal	N14.0-14.3,N15-16,						
sclerosis	N18-19,N26						
**Male**							
MN of urinary organ	C67-C68	4	2.10 (0.57-5.37)	4	2.54 (0.69-6.49)	4	3.93* (1.07-10.39)
MN of kidney & other urinary organs	C64-66,C68	2	2.09 (0.25- 7.53)	2	2.51 (0.30-9.08)	2	3.86 (0.47-13.94)
MN of bladder	C67	2	2.12 (0.26-7.64)	2	2.56 (0.31-9.26)	2	3.99 (0.48-14.41)
Acute glomerulonephritis,	N00,N01.0-01.8,N04,	0	-	0	-	0	-
nephritic syndrome, renal	N14.4,N17						
failure							
Chronic and unspecified	N01.9,N03,N05,N07,	9	2.36* (1.08-4.48)	6	1.85 (0.68-4.03)	3	1.42 (0.29-4.14)
nephritis, renal failure, renal	N14.0-14.3,N15-16,						
sclerosis	N18-19,N26						
**Female**							
MN of urinary organ	C67-C68	5	4.99** (1.62-11.66)	4	4.79*(1.30-12.24)	3	5.47* (1.13-16.00)
MN of kidney and other	C64-66,C68	4	6.48**(1.77-16.58)	3	5.85* (1.21-17.1)	2	6.03 (0.73-21.78)
urinary organs							
MN of bladder	C67	1	2.60 (0.07-14.44)	1	3.10 (0.08-17.21)	1	4.62 (0.12-25.65)
Acute glomerulonephritis,	N00,N01.0-01.8,N04,	1	2.26 (0.06-12.57)	1	2.84 (0.07-15.79)	0	-
nephritic syndrome, renal	N14.4,N17						
failure							
Chronic and unspecified	N01.9,N03,N05,N07,	8	2.45* (1.05-4.82)	5	1.79 (0.58-4.19)	4	2.15 (0.59-5.50)
nephritis, renal failure, renal	N14.0-14.3,N15-16,						
sclerosis	N18-19,N26						

ICD-10, International Classification of Diseases, 10th Revision.

MN: malignancy.

CI: confidence interval.

*: two-tailed *P* < 0.05, **: two-tailed *P* < 0.01.

## DISCUSSION

The role of Chinese herbal drugs in the pathogenesis of kidney disease and urological cancer has attracted much interest in recent years, and virtually nothing is known about the role of Chinese herbs as a risk factor for these diseases. This study provides evidence that Chinese herbalists are at higher risk for developing urological cancers.

In general, Chinese herbalists have a very different lifestyle from the general population. In a case–control study of Taiwanese Chinese herbalists,^22^ the prevalences of cigarette smoking and alcohol drinking among herbalists were 17.1% and 12.1%, respectively, which were much lower than those in Taiwanese workers (26.7% and 17.3%, respectively).^23^ Chinese herbalists were more likely to adopt a healthy lifestyle. However, the significantly higher mortality risk for urological cancers requires our attention. 

We considered other possible causes for the high rate of urological cancers in Chinese herbalists. Cigarette smoking is a major risk factor for urological cancers.^24^ However, the prevalence of herbalists who smoked cigarettes was much lower than that of other Taiwanese workers (as mentioned above).^22^^,^^23^ Therefore, we believe that smoking is not the agent responsible. Long-term use of analgesics is also an important risk factor for urological cancers.^25^ However, Chinese herbalists do not typically prescribe Western medicines, except in cases of severe illness, such as cancer. Indeed, only 2.9% of herbalists reported chronic use of analgesics,^22^ and the prevalence of analgesic use is not likely to be higher in herbalists than in the general population in Taiwan. Use of analgesics is not responsible for the higher rate of urological cancer in herbalists. Arsenic is a carcinogen associated with urological cancer, and is known to be highly concentrated in artesian-well water from some areas where blackfoot disease is endemic.^26^^,^^27^ We checked the addresses of individuals with urological cancers; none lived in regions with contaminated artesian-well water. Thus, drinking arsenic-contaminated water is probably not related to the increased risk. 

After ruling out these risk factors, Chinese herbal drugs—which sometimes contain aristolochic acid (AA)—are the prime culprit responsible for the increased risk for urological cancer in herbalists. AA is now acknowledged as a strong nephrotoxin and carcinogen, and has been implicated in the development of urological cancers. It is derived from an extract of plants from the *Aristolochia*, *Bragantia*, and *Asarum* species, and is a common ingredient in many Chinese herbs, including *Madouling*, *Tianxianteng*, *Qingmuxiang*, *Guangfangji*, *Guanmutong*, and *Xixin*.^28^^–^^31^ In 1993, Vanherweghem et al were the first to report that many young Belgian women on slimming regimens that contained AA later developed progressive renal failure and urothelial cancer.^32^^–^^34^ AA has now been categorized as an IARC group 1 human carcinogen.^35^ Furthermore, evidence shows that Balkan endemic nephropathy, which was believed to be caused by ochratoxin A, may also be caused by AA.^36^^,^^37^ Because many traditional Chinese herbal drugs are made of plants from *Aristolochia*-related species, and herbalists may be chronically exposed to AA either by ingesting herbal drugs or by exposure at work, Chinese herbalists probably experience very high exposure to AA. In Taiwan, the Committee on Chinese Medicine and Pharmacy of the Department of Health issued a regulation prohibiting all herbal drugs containing AA, except *Xixin*. This regulation has been in effect since 2003, but earlier exposure to herbal drugs containing AA may account for the increased risk for urological cancer observed in herbalists.

Chinese herbal drugs may also be contaminated by heavy metals. In Taiwan, raw Chinese herbs are mainly imported from mainland China,^19^ and many Chinese herbs from China are reported to be contaminated by arsenic and other heavy metals.^38^^–^^41^ We suspect that heavy metal contamination (especially arsenic) may also be related to the increased risk for urological cancer in Chinese herbalists.

We noted that female herbalists were at higher risk for urological cancers than males. In a Belgian cohort, all patients with AA-related urothelial carcinoma were women who had taken part in slimming regimens.^33^^,^^42^ Moreover, one report from China noted that 15 of 17 patients with AA-related urothelial carcinoma were women, and apparently all of them were lay people that had ingested Chinese herbs containing AA.^43^ In the rural areas of the Balkans, inhabitants ate food contaminated with AA and developed nephropathy and upper tract urothelial cancer;^44^^,^^45^ women also had a higher risk for urothelial carcinoma in the upper urinary tract.^46^^,^^47^ These findings suggest that women are more susceptible to AA-related urological cancers than men.

In this study, we also found that the SMR for chronic and unspecified nephritis, renal failure, and renal sclerosis were significantly higher in herbalists (SMR = 2.40; 95% CI: 1.40–3.84). When we analyzed by sex, the SMRs for chronic and unspecified nephritis, renal failure, and renal sclerosis were significantly higher in both men (SMR = 2.36; 95% CI: 1.08–4.48) and women (SMR = 2.45; 95% CI: 1.05–4.82). Because we did not have data on the prevalences of diabetes mellitus and hypertension in herbalists, we could not compare these risk factors with the general population. Although we do not wish to infer that AA is the only causative agent, we suspect that chronic use of Chinese herbal drugs plays a role in the increased risk of CKD. This hypothesis is consistent with the findings of Guh et al, who reported that herbal therapy was an important risk factor for CKD in Taiwan.^15^

A major limitation of this study was our inability to measure the length of exposure. We were unable to do so because the database of the Bureau of Labor Insurance does not have information about the particular herbs used and the extent of exposure. Moreover, traditional Chinese herbal drug stores are usually small businesses, and herbalists are required to participate in all steps of herbal drug manufacturing. Often, there are hundreds of different herbs sold in these stores, including herbs that contain AA. In our study, we are unable to conclude that the increased risk of urological cancer in herbalists is a result of occupational exposure, because there is no evidence that AA is absorbed by dermal contact or inhalation of herbal powders. Instead, we believe that the increased risk may be associated with habitual ingestion of Chinese herbal drugs, which is more common among herbalists than the general public.

Other potential limitations are inherent to this type of retrospective mortality study. Because some herbalists began working before 1985, when the Chinese Herbalist Labor Union was not yet established, the actual person-years at risk would be greater than the number reported. If we have earlier employment data and could extend the observation period, there would likely be a greater number of deaths reported among herbalists (the numerator in the SMR calculation). There would also likely be more deaths (the denominator in the SMR calculation) owing to increased person-years at risk. If the degree of exposure of herbalists to Chinese herbal drugs was not significantly different before and after 1985, the SMR would not change markedly.

Another potential confounder is that we do not know whether there were already cases of urological cancer at the time when herbalists joined the union. By adopting a longer lag period (eg, 5 or 10 years), these cases could then be categorized into an unexposed group and would not confound our results. Yet another limitation is that the histological types of urological cancers reported are not known, because the mortality registry database does not specify whether the observed cases of cancer were actually urothelial carcinoma (a specific neoplastic complication of AA exposure) or kidney adenocarcinomas, which are much less likely to be related to AA exposure. Due to these limitations, our results should be interpreted with caution.

Because Chinese medicine has long been common in Taiwan, some Taiwanese people may also have been chronically exposed to Chinese herbal drugs, even though they were categorized as non-exposed. This potential misclassification might result in an underestimation of the risk. As a result, the actual risk among herbalists might be greater than our estimate.

The increased mortality risk for urological cancers and CKD in Chinese herbalists makes assessment of the safety of herbs an urgent necessity. Moreover, members of the general public who habitually use Chinese herbs may have a higher risk than that of Chinese herbalists because they may buy herbs from laypersons or unlicensed herbal stores. In these situations, there are higher risks of misuse, misidentification, and improper preparation and processing due to a lack of professional knowledge. Also, the herbs may come from unknown sources, and adulteration or contamination may be more common.^48^^–^^50^ Therefore, we suggest that the public should receive more and better information regarding the potential adverse effects of herbal drugs.
